# Molecular Functions of Ubiquitin-like Modifiers in Bacterial Infection

**DOI:** 10.3390/cells15121060

**Published:** 2026-06-10

**Authors:** Tohru Tezuka, Wei Jie Nicholas Yang, Keisuke Kitahata, Aya Nohara, Sun Joo Park, Minsoo Kim

**Affiliations:** 1 Laboratory of Integrative Molecular Medicine, Graduate School of Medicine, Kyoto University, Yoshida-konoe-cho, Sakyo-ku, Kyoto-shi, Kyoto 606-8501, Japan; 2 Department of Chemistry, Pukyong National University, Busan 48513, Republic of Korea

**Keywords:** ubiquitin-like proteins, bacterial infection, post-translational modification

## Abstract

Ubiquitin-like proteins (UBLs) such as SUMO, NEDD8, ISG15, FAT10, and UFM1 are proteins that share structural similarities to ubiquitin. Like ubiquitin, they function as protein modifiers, catalyzing modifications through a conserved enzymatic cascade of E1 activating enzymes, E2 conjugating enzymes, and E3 ligases. In doing so, UBLs regulate a diverse set of cellular processes, including stress response, antiviral activity, nuclear transport, cancer development, and autophagy. In recent years, the roles of UBLs during pathogenic bacteria infection have gained attention, although much still remains elusive. This review describes current findings related to UBL systems in the context of pathogenic bacteria infection, focusing on NEDD8, ISG15, FAT10, and UFM1. Specifically, we look at how the host UBL system responds to bacterial infection by inducing the host’s defense system, and how pathogenic bacteria manipulate the host UBL system to ensure successful infection.

## 1. Introduction

Infectious diseases remain among the leading causes of death worldwide; they continue to emerge and re-emerge, occasionally resulting in unpredictable life-threatening epidemics [1]. Antibiotics and antibacterial agents have saved the lives of millions of people, but drug-resistant bacteria continue to increase with their use [2,3]. To overcome pathogenic bacterial infection, we need to better understand the molecular mechanisms of host–bacterial interactions and develop new concepts for antibiotics or vaccines based upon novel mechanisms of action compared to existing antibiotics.

Pathogenic bacteria, such as *Shigella*, *Salmonella*, and *Legionella*, are transmitted to humans by various ways including contact, contaminated water and food, respiratory droplets, and living vectors [4,5,6]. Successful colonization of host cells by pathogenic bacteria depends on the secretion of a variety of toxins and virulence factors, called effectors, into host cells. Often, these toxins/effectors mimic or hijack host proteins and alter host signaling pathways to promote bacterial infection [7,8]. The body deploys multiple defense systems to fight bacterial infection, including epithelial integrity, cell death, quick elimination of infected cells, autophagy, and antimicrobial peptides [9]. In response, bacteria have developed various strategies to evade the host’s defense system. It has been reported that some pathogenic bacteria modulate the host’s ubiquitin system to suppress the host’s defense system and allow establishment of infection [10,11]. In addition to ubiquitination, the role of ubiquitin-like proteins (UBLs) in bacterial infection is emerging. This review describes the impact of bacterial infection on UBL systems, specifically NEDD8, ISG15, FAT10, and UFM1. In particular, we introduce the current understanding of the roles and substrates of these four UBLs specifically in the cellular response to bacterial infection. Specific enzymes that catalyze the ubiquitin system have been successfully developed as drug targets for several diseases [12,13]. Therefore, the characterization of enzymes or substrates that catalyze specific UBL-mediated reactions critical for infection will provide new targets for the development of effective antibacterial drugs.

## 2. Ubiquitin-like Proteins (UBLs) and Their Physiological Role in Bacterial Infection

**Figure 1:**
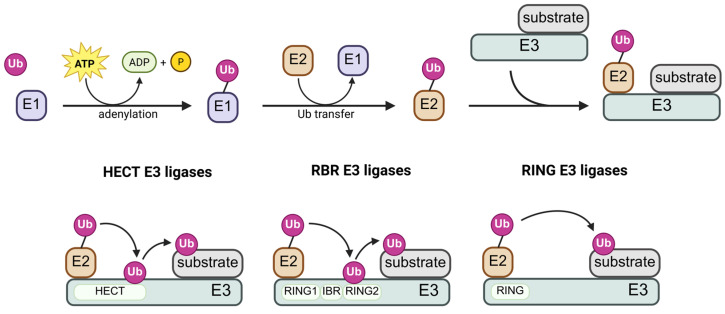
Ubiquitination and the mechanistic differences among E3 ubiquitin ligase families. Ubiquitination proceeds via three enzymatic reactions. However, the last enzymatic reaction involving ubiquitin transfer to the substrate varies depending on the E3 ubiquitin ligase. For HECT E3 ligases and RBR E3 ligases, ubiquitin is first transferred to a cysteine residue on the HECT or RING2 domains respectively, before being transferred to the substrate. For RING E3 ligases, ubiquitin is transferred directly to the substrate from the E2 ubiquitin-conjugating enzyme. E1: ubiquitin-activating enzyme. E2: ubiquitin-conjugating enzyme. E3: ubiquitin ligase.

Ubiquitin (Ub) is first synthesized as a precursor that is processed at the conserved C-terminal Gly76 residue by the ubiquitin carboxy-terminal hydrolases, exposing a diglycine motif that serves as the attachment site for target substrates [14,15]. The covalent attachment of ubiquitin to the substrates (ubiquitination) requires three enzymatic reactions. First, the exposed C-terminal glycine of Ub is adenylated by the E1 ubiquitin-activating enzyme (UBA1 and UBA6) in an ATP-dependent manner and then transferred to the E1 cysteine side chain via a thioester linkage [15]. Activated Ub is subsequently transferred to the ubiquitin-conjugating enzymes (E2), forming another thioester linkage. Finally, ubiquitin ligase (E3) transfers Ub to the Lys residue on substrates, forming an isopeptide bond (Figure 1). Ubiquitination is reversible, and deubiquitinating enzymes (DUBs) catalyze the removal of ubiquitin moieties from substrates [16].

Protein substrates may be mono-ubiquitinated when a single Ub molecule is attached to the Lys residue on the substrate, multi-ubiquitinated when multiple Ub molecules are attached to different Lys residues on the substrate, or poly-ubiquitinated when additional Ub molecules are further attached to a pre-existing Ub molecule on the substrate [17,18]. In the case of poly-ubiquitination, different topologies of polyubiquitin chains can be generated. Ub has seven Lys residues at residues 6, 11, 27, 29, 33, 48, and 63, to which additional Ub molecules can be attached. For instance, Lys11- or Lys48-linked polyubiquitin chains lead to proteasomal degradation, while Lys63-linked polyubiquitin chains are primarily involved in non-proteolytic processes such as DNA repair, signal transduction, and protein trafficking [18]. In addition, linear or Met1-linked polyubiquitin chains, which are generated by the Linear Ubiquitin Chain Assembly Complex (LUBAC), are important in the regulation of NF-κB activation and xenophagy [19,20]. Hence, although protein ubiquitination serves as a molecular tag of proteasomal degradation, it also affects a wide range of cellular processes including cell signalling, cell cycle, and inflammatory responses [21,22,23].

**Figure 2:**
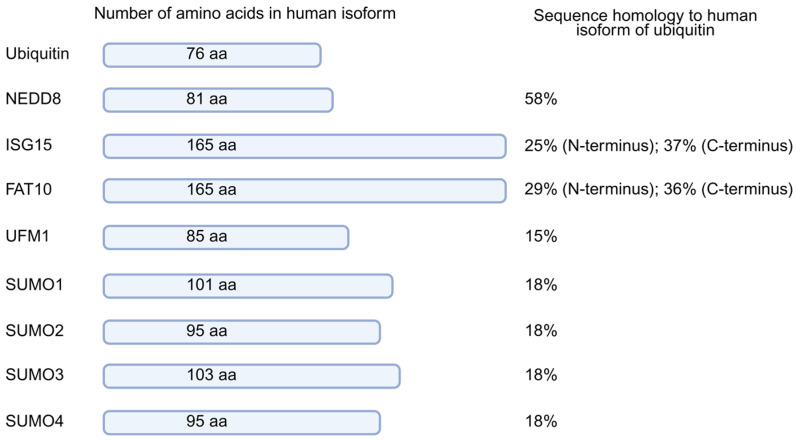
The human isoforms of ubiquitin and the ubiquitin-like proteins (UBLs) NEDD8, ISG15, FAT10, UFM1, and SUMO1–SUMO4. These UBLs share low amino acid sequence homology to ubiquitin, ranging from 15% in UFM1 to 58% in NEDD8.

UBLs share low sequence similarity with Ub, but possess the same ubiquitin structural fold (Figure 2) [14,24,25]. UBLs are conjugated to their substrate proteins via an entirely conserved enzymatic cascade. The conjugation reaction cascades resemble those of the ubiquitin system, and a few of the cascade enzymes have been identified (Table 1) [14,24,25]. Compared to ubiquitination, modification by UBLs is less understood and few of their substrates have been identified [25,26]. Several UBL family proteins have been identified in eukaryotes, including SUMO, NEDD8, ISG15, FAT10, ATG8, ATG12, URM1, UFM1, UBL5, FUB1, and DWNN [24,26,27]. In prokaryotes, ubiquitin-like proteins such as SAMP and PUP have also been identified and reviewed [28,29]. There are many reviews describing the regulation of autophagy and bacterial infection by ATG8 and ATG12 [30,31,32], as well as the role of SUMOylation in bacterial infection [33,34,35], and thus they will not be presented here. Instead, we focus on the host’s UBL pathways such as NEDDylation, ISGylation, FAT10ylation, and UFMylation, whose role in bacterial infection has been made clearer in recent years (Table 2).

### 2.1. Neuronal Precursor Cell-Expressed, Developmentally Down-Regulated Gene 8 (NEDD8)

Among the UBLs, NEDD8 shows high similarity (58% identity) to ubiquitin (Figure 2) [25,36]. The C-terminus of NEDD8 is processed by NEDP1 (DEN1, SENP8) or UCHL3 to generate the mature form of NEDD8 [36,37]. NEDD8 is covalently linked to substrates through a cascade of reactions involving E1 (APP-BP1 and UBA3), E2 (UBE2M or UBE2F), and NEDDylation E3 ligases. More than a dozen of NEDDylation E3 ligases have been identified, and all of them are dual E3 ligases that can catalyze both NEDDylation and ubiquitination [38]. The well-studied and most abundant NEDD8 substrates are the Cullins, which are scaffold proteins for the largest class of ubiquitin E3 ligases, termed CRLs (Cullin-RING ubiquitin ligases). Cullins are NEDDylated by the RING-type E3 ligase RBX1 (or RBX2), which works synergistically with the DCNL family of co-ligases to facilitate efficient NEDD8 conjugation. NEDDylation of Cullins increases the ubiquitin ligase activity of CRLs by triggering structural changes. NEDD8 is deNEDDylated from Cullin by the COP9 signalosome (CSN) [36,39]. In recent years, various non-Cullin NEDD8 substrates, including p53, MDM2 (murine double minute 2), and EGFR (epidermal growth factor receptor), as well as their corresponding E3 ligases, have been identified [36,40,41]. Therefore, NEDDylation is critical for cell proliferation, cancer progression, and apoptosis. Poly-NEDDylation may also exist, although its function is not known [25].

**Figure 3:**
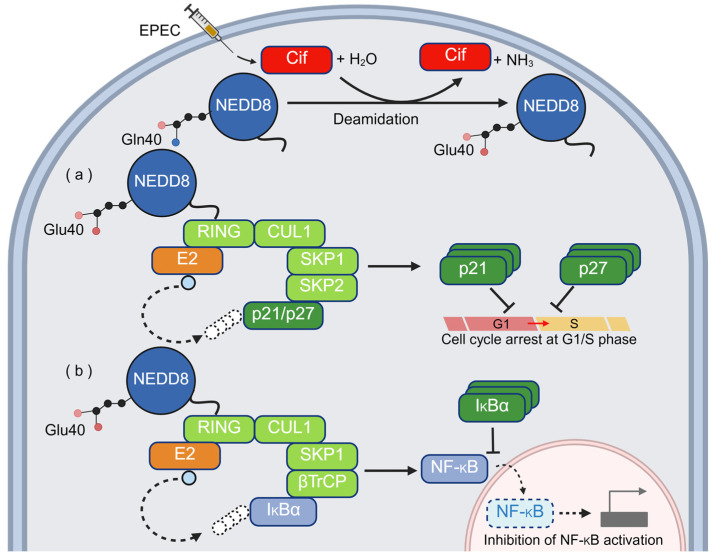
The Cif family of type III effector proteins are responsible for the deamidation of Gln40 on NEDD8 to Glu40, leading to the suppression of CRL activity. (**a**) Deamidation of NEDD8 leads to the accumulation of CRL substrates such as p27^Kip1^ and p21^Cip1^ and subsequent cell cycle arrest at the G1/S phase. (**b**) Deamidation of NEDD8 may also lead to inhibition of the CRL-dependent degradation of IκBα, and thus the inhibition of NF-κB activation.

Some pathogenic bacteria manipulate the host NEDDylation pathway by secreting effector proteins into the host cells [25]. The cycle-inhibiting factors (Cifs) family of type III effector proteins are deamidases that are conserved in Gram-negative pathogenic bacteria such as enteropathogenic *Escherichia coli* (EPEC), enterohemorrhagic *Escherichia coli* (EHEC), *Yersinia pseudotuberculosis*, *Photorhabdus luminescens*, and *Burkholderia pseudomallei* [42,43]. Crystallographic analyses have shown that Cif family proteins contain a Cys–His–Gln catalytic triad and deamidate a specific glutamine residue in their substrates [44,45,46,47,48]. The Cif of EPEC (CifEc) recognizes its host target, NEDD8, and specifically deamidates Gln40 to Glu40. Deamidation of NEDD8 inhibits the activity of CRLs, leading to the accumulation of CRL substrates such as p27^Kip1^ and p21^Cip1^, which are cell cycle inhibitors, during EPEC infection. Consequently, EPEC-infected cells undergo cell cycle arrest (Figure 3a) [49,50,51]. The intestinal epithelium self-renews every few days, and the rapid turnover of gut epithelial cells is a host defense system against bacterial infection [9]. Cif-induced cell cycle arrest may contribute to the slowdown of this host self-renewal process. The Cif homolog in *Burkholderia pseudomallei* (CHBP) deamidates Ub and NEDD8 [47,49]. NEDD8 deamidation catalyzed by CHBP is 10-fold more efficient than Ub deamidation [47]. The crystal structures of CHBP in complex with NEDD8 or Ub have suggested that Ub is more mobile than NEDD8 in the CHBP complex. The important amino acids for substrate recognition are conserved in Ub and NEDD8, but not in other ubiquitin-like proteins. Deamidation of NEDD8 by CHBP induces macrophage-specific apoptosis [47]. Cif inhibits both TNFα and *Burkholderia thailandensis*-induced NF-κB activation, partially by inhibiting the CRL-dependent degradation of IκBα (Figure 3b) [52]. A recent structural study solved the long-time question regarding how deamidation of NEDD8 suppresses the activity of CRLs by revealing that the Glu40 modification destabilizes the activation form of the CRL complex, resulting in CRL being unable to conjugate ubiquitin to its substrates [53]. In addition, bacterial-induced oxidative stress targets Cullin for NEDDylation [54,55]. Since there exists hundreds of distinct CRL ligases and CRL substrates in eukaryotic cells, such as Nrf2, HIF-1α, and Cdt1, there remains many unknown roles of NEDD8 in bacterial infection.

**Figure 4:**
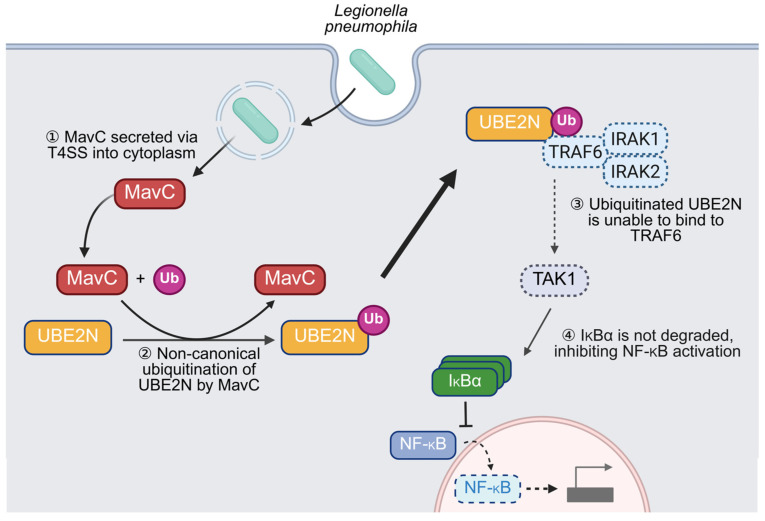
The Legionella pneumophila MavC effector ubiquitinates UBE2N via a transglutamination reaction, leading to the inhibition of downstream NF-κB activation. UBE2N is a host E2 enzyme which associates with TRAF6 (a host E3 ligase) in the NF-κB signaling pathway. Ubiquitination of UBE2N by MavC prevents UBE2N from binding to TRAF6, thereby inhibiting further downstream activation of the NF-κB pathway.

An interesting structural homolog of Cif and CHBP is MavC, a *Legionella pneumophila* effector protein. NMR analyses have shown that MavC possesses a main deamidase domain containing the Cys–His–Gln catalytic triad similar to Cif and CHBP, but possesses an additional insertion domain which is otherwise absent in Cif and CHBP [56]. Despite being structural homologs, MavC has been shown to function as a deamidase specific for ubiquitin but not NEDD8, and also to catalyze the non-canonical ubiquitination of UBE2N [56,57]. The incompatibility of NEDD8 binding to MavC could be due to steric and electrostatic clashes between the side chain of Lys22 on NEDD8 and the side chain of Lys148 in MavC [58]. Mechanistically, the non-canonical ubiquitination of UBE2N has been proposed to proceed via a transglutamination reaction, whereby Cys74 on MavC first launches a nucleophilic attack on Gln40 in ubiquitin to form a thioester intermediate, which subsequently reacts with the amine side group in Lys92 (the major modification site) or Lys94 of UBE2N to form an intermolecular isopeptide bond [57]. Unlike canonical ubiquitination which requires host E1 and E2 enzymes, MavC has been shown to be able to do so in the absence of such host enzymes [57]. In the absence of a suitable amine donor, however, the γ-glutamylthioester bond formed between Cys74 of MavC and Gln40 ubiquitin is hydrolyzed to form Glu40 on ubiquitin, resulting in net ubiquitin deamidation [57]. It is important to note, however, that the main function of MavC is likely to be that of UBE2N ubiquitination instead of ubiquitin deamidation; in cells infected with wild-type *L. pneumophila*, only ubiquitinated UBE2N, but not deamidated ubiquitin, was detected [57], and biolayer interferometry experiments revealed that MavC’s affinity for free ubiquitin was too low for a meaningful level of ubiquitin deamidation under cellular conditions [59]. UBE2N ubiquitination by MavC has been shown to abolish its E2 activity and dampen the NF-κB pathway in the early phase of *L. pneumophila* infection (Figure 4), with its effect in inhibiting NF-κB activation demonstrated to be comparable to that of Cif from *Yersinia pseudotuberculosis* [56,57]. In the later phases of *L. pneumophila* infection, MvcA, a close structural homolog of MavC also originating from *L. pneumophila*, functions as a regulator of MavC by removing ubiquitin from UBE2N, allowing the activation of the NF-κB pathway to facilitate robust intracellular bacterial replication [60].

There have been several studies on how NEDDylation affects ubiquitination in the absence of pathogenic bacteria infection. An example of ‘positive crosstalk’ was shown in a study which demonstrated how exercise and activation of cAMP signaling led to increased NEDDylation of E3 ligases, leading to an increase in global protein ubiquitination [61]. Other studies showed ‘antagonistic crosstalk’, whereby the NEDDylation of certain proteins were crucial in preventing its ubiquitination. The NEDDylation of LC3B was shown to reduce LC3B’s interaction with E3 ligases VHL and BIRC6, thereby decreasing the LC3B ubiquitination [62]. In the pathogenesis of renal fibrosis in diabetic nephropathy, NEDDylation of RhoA by upregulated NAE1 inhibited the ubiquitination and degradation of RhoA [63]. However, studies on NEDDylation and ubiquitination in the context of pathogenic bacteria infection remain scarce. Within the context of the Cif family of bacteria effector proteins, a study showed how infection of host cells by *Yersinia pseudotuberculosis* or EPEC leads to the entry of Cifs which deamidate NEDD8, impairing CRL activity and blocking ubiquitin-dependent trafficking of Perforin-2 [64]. Future studies investigating changes in the host cell NEDDylome and ubiquitome before and during pathogenic bacteria infection could offer clearer insights into the crosstalk between NEDDylation and ubiquitination and how it enables pathogenic bacteria to manipulate the host cell machinery to establish infection.

### 2.2. Interferon-Stimulated Gene 15 (ISG15)

The type I interferon (IFN)-inducible protein, ISG15, consists of two ubiquitin-like domains connected by a short linker, with a conserved C-terminal LRLRGG sequence [25,26,65]. The N- and C-terminal domains share 25% and 37% homology to ubiquitin, respectively (Figure 2). To date, ISG15 has been found only in higher eukaryotes and is absent in yeast, *Caenorhabditis elegans*, and *Drosophila melanogaster*, implying that ISG15 is involved in the IFN signaling pathway, which mediates the innate immune response to viral infections [24,25,65]. Human ISG15 shows only ~63% identity to mouse ISG15, suggesting functional diversity between these two species. Like ubiquitination, conjugation of ISG15 (ISGylation) requires E1 (UBA7), E2 (UBE2L6), and several E3 ligases [66]. The homologous to the E6-AP carboxyl terminus (HECT)-type E3 ligase Herc5, is a principal ISG15-specific E3 ligase, although two other E3 ligases, TRIM25/EFP and HHARI, are reported to catalyze ISGylation under certain conditions [67,68,69].

Ubiquitin-specific protease 18 (USP18) has been identified as an ISG15 deconjugation enzyme [70]. Notably, expression of UBA7, UBE2L6, HERC5, and USP18 were elevated following treatment with type I IFNs (IFNα and IFNβ) [25,71]. ISGylation is not a signal for proteasomal degradation, and while multi- and mono-ISGylation has been reported, poly-ISGylation has not [25,72]. ISG15 targets both host and viral proteins, including key regulators of signal transduction (for example, Janus-activated kinase 1 (JAK1), signal transducers and activators of transcription 1 (STAT1), interferon regulatory factor 3 (IRF3), and retinoic acid-inducible gene-1 (RIG-1)), newly synthesized proteins associated with polyribosomes, and nascent viral proteins [66,73,74]. Therefore, ISGylation plays an important role in the innate immune response to viruses and cell surface trafficking of cellular/viral proteins, including glycoproteins and cytokines [75,76].

ISGylation may interfere with the ubiquitin–proteasome pathway in multiple ways, such as through the inhibition of E2 for ubiquitination and through competition between ISGylation and ubiquitination on common modification sites. E2 enzyme UBE2N, which catalyzes the attachment of a Lys63-linked polyubiquitin chain with a specific E3 ligase (TRAF6) and thereby activating the host inflammatory response [77], can be modified by ISG15. ISGylation of UBE2N suppresses its ability to form Ub thioester bonds [78,79]. Elevated expression and conjugation levels of ISG15 in cancer cells of different origins decrease polyubiquitination, suggesting that ISGylation may negatively regulate ubiquitination [80].

**Figure 5:**
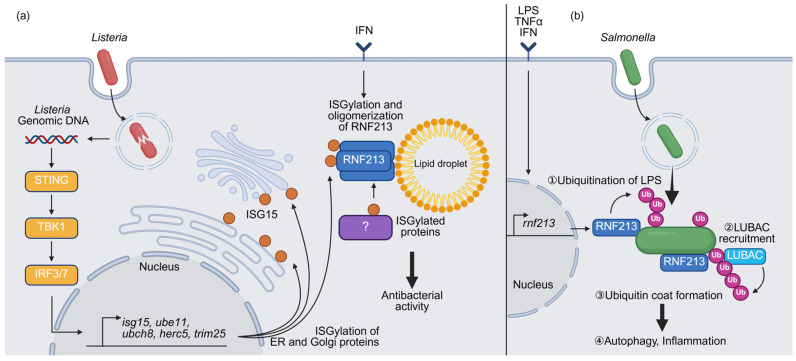
The roles of ISG15 and RNF213 in *Listeria* and *Salmonella* infection. (**a**) During *Listeria* infection, its genomic DNA induces STING–TBK1-dependent IRF3 and IRF7 signaling (CSP). IRF3/7 activation induces the expression of ISGylation-related enzymes and inflammatory cytokines. An increase in these proteins results in ISGylation of ER and Golgi proteins. Moreover, IFN induces ISGylation and oligomerization of RNF213 on lipid droplets, where it acts as a sensor for ISGylation. (**b**) During *Salmonella* infection, RNF213 induces LPS ubiquitination which results in the recruitment of LUBAC. RNF213 and LUBAC form a ubiquitin coat on *Salmonella*, inducing autophagy and inflammation.

The role of ISG15 in viral infection is particularly well studied [71,81]; however, its role during bacterial infection has not been fully elucidated. Exposure to type I IFNs, LPS, double-stranded RNA, and genotoxic stress induce the expression of ISG15 and ISGylation [25,74,82]. Pioneering studies have provided insights into the regulation of ISGylation during *Listeria* infection [75,83]. ISG15 can be induced by *Listeria* in an interferon-independent manner in human nonphagocytic cells. *Listeria* genomic DNA is sensed and activates the STING/TBK1/IRF3/IRF7 pathway, named the cytosolic surveillance pathway (CSP). CSP directly induces ISG15 and ISGylation-related enzymes, leading to ISGylation of ER and Golgi proteins, such as RTN4 and magnesium transporter 1 (MAGT1). ISG15 modification of distinct ER and Golgi proteins increases secretion of cytokines during *Listeria* infection (Figure 5a). ISG15-deficient fibroblasts are highly susceptible to *Listeria* infection [75]. Mice bearing catalytically inactive USP18 display hyper-ISGylation following *Listeria* infection; this hyper-ISGylation protects against *Listeria* infection [83].

Recently, RING Finger Protein 213 (RNF213), a giant E3 ubiquitin ligase, was discovered to be a key antimicrobial protein regulating ubiquitination and ISGylation. RNF213 contains a dynein-like ATPase core and a multidomain E3 module [84,85]. RNF213 is involved in inflammatory and immune responses, with its expression upregulated by signals such as LPS, TNFα, and interferon [86]. RNF213 was identified to ubiquitinate the lipid A moiety of LPS during *Salmonella* infection, demonstrating a novel non-proteinaceous substrate ubiquitination [85]. RNF213-mediated LPS ubiquitination subsequently recruits LUBAC and induces linear ubiquitination in the bacterial ubiquitin coat. RNF213-deficient cells fail to generate a bacterial ubiquitin coat, induce anti-bacterial autophagy, and limit *Salmonella* proliferation, demonstrating RNF213’s critical antibacterial function (Figure 5b) [85]. RNF213 also plays an important role in antimicrobial activity against other bacteria such as *Chlamydia trachomatis* [87] and *Listeria monocytogenes* [88], several viruses [88], and the intracellular protozoan parasite *Toxoplasma gondii* [89].

More interestingly, RNF213 was identified as the most ISGylated protein upon *Listeria* infection [83]. RNF213 was also identified as an ISG15-binding protein by Virotrap, a new mass spectrometry-based strategy for analyzing protein complexes [88,90]. Further analyses showed that IFN induces ISGylation and subsequent oligomerization of RNF213 on lipid droplets (LDs), where it binds to ISGylated proteins, thereby acting as a sensor for ISGylation. RNF213-deficient mice are susceptible to *Listeria* infection, highlighting the importance of RNF213’s E3 ligase activity for antimicrobial activity [88]. LDs are dynamic organelles that, apart from regulating energy homeostasis through lipid storage and hydrolysis, also play important roles as intracellular innate immune hubs [91]. To understand the roles of the functional interaction between RNF213 and ISG15 in antimicrobial responses, it is critical to uncover intracellular signals that are evoked by RNF213 ISGylation, RNF213 oligomerization, and binding of RNF213 with ISGylated proteins.

Illustrating a bacterial strategy to counteract host RNF213-mediated defenses, recent studies revealed that *Shigella flexneri* evades host RNF213-mediated LPS ubiquitination through IpaH1.4 [92,93]. IpaH1.4 belongs to the IpaH family of effector proteins found in *Shigella* that are delivered into host cells via the type III secretion system, functioning as a NEL-type bacterial E3 ligase [94]. HeLa cells infected with wild-type *Shigella* showed depleted levels of endogenous RNF213, but this was not observed upon infection with *Shigella* strains lacking IpaH1.4. Additionally, LPS ubiquitination was not observed in cells infected with wild-type *Shigella*, but was present in cells infected with *Shigella* strains lacking IpaH1.4. Structural data obtained from cryo-EM and X-ray crystallography showed that the canonical concave substrate-binding site of the leucine-rich repeat (LRR) domain of IpaH1.4 recognizes and binds specifically to the RING domain of RNF213, thereby occluding the E2-binding face of the RING domain [92,93]. Consequently, IpaH1.4 catalyzes Lys48-linked ubiquitination of RNF213 through the canonical ubiquitination mechanism, targeting it for proteasomal degradation. Notably, further unbiased pulldown mass spectrometry screens performed using HeLa cell lysates to detect IpaH1.4 interactors found enrichment for six E3 ligases involved in inflammatory responses, of which five contain a RING domain (TRIM25, TRIM47, DTX3L, TRAF2, and BIRC3) and one contains a B-box-type Zn finger (TRIM29) [92]. As described above, TRIM25 is reported to be involved in ISGylation [95]; therefore, the results of the mass spectrometry screen imply potential crosstalk of IpaH1.4 indirectly affecting the ISGylation of proteins during infection.

ISG15 is also present in the medium of IFN-treated lymphocytes and monocytes and functions as a secreted molecule [96]. Mendelian susceptibility to mycobacterial disease (MSMD) is a rare genetic disease caused by genetic deficiencies in genes *IFNGR1*, *IFNGR2*, *STAT1*, *IL12b*, *IL12RB1*, *NEMO*, *CYBB*, *IRF8*, and *ISG15*. MSMD patients are highly sensitive to infection with weakly virulent mycobacteria, including environmental mycobacteria and *Mycobacterium bovis* Bacille Calmette-Guérin (BCG) vaccine, but not some viral infections [96,97]. MSMD patients who have null mutation in ISG15 are also susceptible to *Salmonella* and *Mycobacterium tuberculosis* infection due to the loss of secreted ISG15 [97,98]. Like humans, ISG15-deficient mice are also more susceptible to mycobacterial infections. The lack of secreted ISG15 during mycobacterial infection leads to impaired IFN-γ secretion by natural killer (NK) cells, and the addition of ISG15 restores IFN-γ levels, suggesting that secreted ISG15 plays an essential role in anti-mycobacterial immunity [97]. Recently, leukocyte function-associated antigen-1 (LFA-1) integrin has been identified as a receptor of secreted ISG15 in NK cells [99].

Free ISG15 binds ubiquitin-specific peptidase 18 (USP18), preventing SKP2-mediated degradation of USP18. Accumulation of intracellular USP18 leads to a decrease in IFN-induced signaling. ISG15-deficient patients have lower protein levels of USP18 due to increased degradation of USP18, thereby inducing strong IFN-α/β immunity [100]. IFN-α/β amplification explains the lack of viral infection phenotypes in patients with ISG15 deficiency. However, murine ISG15 does not interact with USP18 and does not control USP18 stability [101]. The susceptibility of ISG15-deficient mice to several viral infections, but not humans, may be due to different biochemical properties of murine versus human ISG15. In addition, ISG15 is a complex and multifunctional protein that affects the immune system [65]. ISG15-deficient, but not UBA7-deficient, mice are susceptible to Chikungunya virus infection, suggesting that conjugated or unconjugated ISG15 has different antiviral responses [102]. Many questions remain to be elucidated, including how ISG15 inhibits USP18 proteolysis, how ISG15 is secreted, and how secreted ISG15 stimulates the IFN-γ induction pathway.

### 2.3. HLA-F Adjacent Transcript 10 (FAT10)

FAT10, also known as ubiquitin D (UBD), is a vertebrate-specific UBL possessing two UBL domains [103]. The N- and C-terminal ubiquitin-like domains share 29% and 36% homology to ubiquitin, respectively (Figure 2) [104]. Unlike ubiquitin, which needs to be processed by a specific protease from their inactive precursors, FAT10 is synthesized as a mature protein with a terminal diglycine motif [24].

While it is largely established that FAT10 covalently binds to its target molecules through the activity of E1 (UBA6) and E2 (USE1) enzymes [105,106], a recent study has, through transcriptomic analyses and biochemical screenings, identified additional E2 enzymes capable of accepting UBA6-activated FAT10 in the absence of USE1 [107]. To date, Parkin has been identified as the only FAT10 E3 ligase that FAT10ylates Mitofusin-2 during mitophagy [108]. Several substrates of FAT10ylation such as p62/SQSTM1, HUWE1, and UBA1 have been reported [109,110], but their respective E3 ligases remain unidentified. No FAT10 deconjugating enzymes have yet been identified [111].

FAT10ylation is the only modification apart from ubiquitination capable of inducing proteasomal degradation of target proteins [112]. Unlike ubiquitin, which is deconjugated from its substrates by deubiquitinating enzymes prior to proteasomal degradation of its substrates, FAT10 is not deconjugated but instead degraded along with its substrates, leading to FAT10 possessing a short half-life [111]. FAT10 is highly expressed in immune tissues such as the thymus and secondary lymphatic organs; its expression in other tissues and cells is induced by inflammatory cytokines including TNFα and IFNγ, and is upregulated during dendritic cell maturation [113]. Moreover, FAT10 expression is increased in various cancers, liver cirrhosis, and HIV-associated nephropathy [114,115,116]. FAT10-deficient lymphocytes undergo spontaneous apoptosis and are highly susceptible to endotoxin (LPS) administration [117]. FAT10ylation has been implicated in various cellular responses, including antigen presentation, cell cycle progression, and the NF-κB signalling pathway [113,114]. Collectively, these observations suggest the involvement of FAT10 in inflammation and tumorigenesis.

Autophagy plays a critical role in the clearance of invading bacteria [30,31,118]. In contrast to bacteria like *Listeria*, which utilises ActA to manipulate host cell actin to evade FAT10 decoration and autophagy [119], *Salmonella* invading the cytoplasm is coated with FAT10 and co-localizes with autophagy marker molecules including p62, NDP52, and LC3B. When FAT10-deficient mice are infected with *Salmonella*, the number of colonised bacteria is higher than that in wild-type mice, resulting in higher mouse mortality [120]. These observations suggest that FAT10ylation targets *Salmonella* selectively to autophagy, contributing to bacteria clearance. Further studies are desperately needed to fully elucidate the physiological role of FAT10ylation in pathogenic bacteria infection.

### 2.4. Ubiquitin-Fold Modifier 1 (UFM1)

UFM1 shares low sequence identity with Ub (~15%), and its mechanism of action is conserved in most eukaryotes but not in yeasts (Figure 2) [121]. Newly synthesized pro-UFM1 is cleaved by UFSP1/2 at its C-terminal Ser-Cys dipeptide, thereby exposing a C-terminal Gly residue necessary for conjugation [122]. UFSPs also act as de-UFMylation enzymes [122]. UFM1 and the E2 enzyme UFC1 have been identified as E1 enzyme UBA5-associated proteins by mass spectrometry analysis [123], with UBA5 demonstrated to activate UFM1 via a two-step mechanism involving a binary covalent complex of UBA5~UFM1 thioester [124].

UFL1 is the only known E3 ligase of UFMylation [125]. Because the UFM system is essential for tissue homeostasis and development, UFM component deletion mice show embryonic lethality [126]. The UFM system is also tightly associated with endoplasmic reticulum (ER) proteostasis [121]. One of the most well-known UFMylation substrates, UFBP1, recruits UFL1 to the ER surface and induces UFMylation of the 60S ribosomal protein L26 and ribophorin I, eventually facilitating autophagy of the ER (ER-phagy) [127].

Although the function of UFMylation in bacterial infections is not yet fully understood, the expression level of UFM1 increases upon LPS stimulation of human endothelial cells and UFM1 inhibits the NF-κB signaling pathway [128]. Proteomic analysis shows that LLO treatment decreases UFC1 levels in HeLa cells, implying that UFMylation can be deregulated by *Listeria* during infection [129]. Collectively, these reports suggest a possible role of UFMylation as a defense mechanism against bacterial infection.

## 3. Conclusions and Future Prospects

Diverse proteomic studies revealed crosstalk between ubiquitin, UBLs, and other PTMs such as phosphorylation and acetylation [130]. For instance, many SUMOylated lysins have also been found to be ubiquitinated, acetylated, or methylated, thus indicating crosstalk between PTMs and UBLs [131]. Quantitative proteomic analysis following *Listeria* infection shows significant overlap with both ubiquitination and ISGylation, suggesting competition for specific lysines on their substrates [83]. LLO decreases E2 protein levels including UBE2N, UBE2K, and UBE2I, and it alters the host proteome including NEDDylation and ubiquitination [129]. In addition, hybrid chains with Ub and UBLs, such as Ub-NEDD8, Ub-SUMO, and Ub-ISG15, are generated under stress or physiological conditions [132]. Hybrid chain formation must be tightly regulated in a spatial and temporal manner; thus, decoding the ubiquitin and UBL crosstalk is critical to understanding cellular defense processes.

Pathogenic bacteria can sense and respond to various host defense responses and microenvironmental stresses encountered during infection. These responses trigger pathogens to induce virulence-associated effector proteins to overcome the antibacterial response. Considering that extensive crosstalk exists between UBLs and ubiquitin, it is paramount that new bacterial effectors capable of targeting or antagonizing host ubiquitin or UBL modifications be identified, as these would provide novel insights into the nature of the UBL system, mechanisms of virulence against host defense systems, and potential targets for the prevention or treatment of infectious disease. Beyond the scope of infectious diseases, links to other human diseases, such as cancer, may be elucidated as well, leading to potential new therapies for the treatment of such diseases.

## Figures and Tables

**Table 1:** Overview of ubiquitin and UBL modification systems.

UBL	Activating Enzyme	Conjugating Enzyme	Ligase	Substrate	Deconjugation
Ubiquitin	UBA1, UBA6	UBE2 family	HECT, RBR, RING	>10,000	DUBs
SUMO1–4	AOS1-UBA2	UBE2I	PIAS family	>1000	SENPs
NEDD8	APPBP1-UBA3	UBE2F, UBE2M	RBX1/2-containing Cullin complexes/DCN1 family	Cullins, etc.	NEDPs
ISG15	UBA7	UBE2L6	HERC5	JAK1, STAT1, IRF3, RIG-1, etc.	USP18
FAT10	UBA6	USE1, etc.	Parkin	p62, HUWE1, UBA1, etc.	unknown
UFM1	UBA5	UFC1	UFL1	a few	UFSP1/2

Six classes of UBLs and their enzymes participate in UBL conjugation/deconjugation systems. Like ubiquitin, a UBL is attached to their substrate through the three-enzyme cascade, and each UBL deconjugation enzyme removes UBL from the substrates, though the exact deconjugation enzyme has yet to be elucidated for FAT10. Each enzyme and UBL are structurally similar but are involved in various biological pathways. PIAS is a Siz/PIAS RING (SP-RING) type E3 ligase. Rbx1/2 are RING type E3 ligases. HERC5 is a HECT type E3 ligase. UFL1 has no sequence similarity with E3 ligases from other ubiquitin systems, instead acting as a scaffold-like E3 ligase.

**Table 2:** The ubiquitin or UBL modifications and consequences upon pathogenic bacteria infection. EPEC: enteropathogenic *Escherichia coli*. EHEC: enterohaemorrhagic *Escherichia coli*.

Pathogenic Bacteria	Bacterial Component	Ubiquitin or UBL Modification	Consequences
*Shigella*	IpaH1.4	RNF213 ubiquitination (Lys48)	Proteasomal degradation of RNF213 and suppression of inflammation
EPEC/EHEC	Cif	NEDD8 and Ub deamidation	Inhibition of CRL ubiquitin ligases
*Burkholderia pseudomallei*	CHBP	NEDD8 and Ub deamidation	
*Photorhabdus luminescens*	Cif_Pl_	NEDD8 deamidation	
*Yersinia pseudotuberculosis*	Cif_Yp_	NEDD8 and Ub deamidation	
*Legionella pneumophila*	MavC	Ub deamidation and UBE2N non-canonical ubiquitination (transglutamination)	Inhibition of NF-κB-dependent inflammation
*Listeria*	Genomic DNA	ISGylation of ER and Golgi proteins	Increased secretion of inflammatory cytokines
*Salmonella*	LPS	Ubiquitination of LPS by RNF213; induction of linear ubiquitination of bacterial ubiquitin coat by LUBAC	Autophagy and inflammation

## Data Availability

No new data were created or analyzed in this study.

## Abbreviations

The following abbreviations are used in this manuscript:

ActA	Actin assembly inducing protein
AOS	Activator of SUMO
APP-BP1	Amyloid precursor protein-binding protein 1
ATG	Autophagy-related protein
BCG	Bacille Calmette-Guérin
BIRC3/6	Baculoviral IAP repeat-containing protein 3/6
cAMP	Cyclic adenosine monophosphate
Cdt1	Chromatin Licensing and DNA Replication Factor 1
CHBP	Cif homolog in *Burkholderia pseudomallei*
Cif	Cycle-inhibiting factor
COP9	Constitutive photomorphogenesis 9
CRL	Cullin-RING Ubiquitin Ligase
CSP	Cytosolic surveillance pathway
CYBB	Cytochrome B-245 Beta Chain
DEN1	Deneddylase-1
DTX3L	Deltex E3 Ubiquitin Ligase 3L
DUB	Deubiquitinating enzyme
DWNN	Down with no name
EFP	Estrogen-responsive finger protein
EGFR	Epidermal growth factor receptor
EHEC	Enterohemorrhagic *Escherichia coli*
EPEC	Enteropathogenic *Escherichia coli*
ER	Endoplasmic reticulum
FAT10	F-adjacent transcript 10
FUB1	Fan Ubiquitin-like protein 1
HECT	Homologous to the E6-AP Carboxyl Terminus
HERC	HECT domain and RCC1-like domain Containing Protein
HHARI	Human homolog of Drosophila Ariadne
HIF-1α	Hypoxia-Inducible Factor-1 Alpha
IFN	Type I interferon
IFNGR	Interferon Gamma Receptor
IL12b	Interleukin-12 subunit beta
IL12RB1	Interleukin 12 Receptor Subunit Beta 1
IRF	Interferon regulatory factor
ISG15	Interferon-stimulated gene 15kDa
IκBα	Inhibitor of nuclear factor kappa B alpha
JAK1	Janus kinase 1
LC3B	Microtubule-associated protein 1 light chain 3 beta
LDs	Lipid droplets
LFA-1	Leukocyte function-associated antigen-1
LLO	Listeriolysin O
LPS	Lipopolysaccharide
LRR	Leucine-rich repeat
LUBAC	Linear Ubiquitin Chain Assembly Complex
MAGT1	Magnesium transporter 1
MDM2	Mouse double minute 2 homolog
MSMD	Mendelian subsceptibility to mycobacterial disease
NAE1	NEDD8 Activating Enzyme E1 Subunit 1
NDP52	Nuclear Domain 10 Protein 52
NEDD8	Neural precursor cell expressed developmentally down-regulated protein 8
NEDP	NEDD8-specific peptidase
NEL	Novel E3 Ligase
NEMO	NF-kappa-B essential modulator
NF-κB	Nuclear factor kappa B
NK	Natural killer
Nrf2	Nuclear factor erythroid 2-related factor 2
PIAS	Protein inhibitor of activated STAT
PUP	Prokaryotic ubiquitin-like protein
RBR	RING-in between-RING
RBX1/2	Ring box protein 1/2
RhoA	Ras homolog family member A
RING	Really Interesting New Gene
RNF213	RING finger protein 213
RTN4	Reticulon 4
SAMP	Small Archaeal Modifier Proteins
SENP	SUMO-specific peptidase
SKP2	S-phase kinase-associated protein 2
STAT1	Signal Transducer and Activator of Transcription 1
STING	Stimulator of interferon gene
SUMO	Small ubiquitin-like modifier
TBK1	Tank-Binding-Kinase 1
TNFα	Tumor necrosis factor alpha
TRAF2/6	TNF receptor-associated factor 2/6
TRIM25	Tripartite motif containing protein 25
TRIM47	Tripartite motif containing protein 47
Ub	Ubiquitin
UBA	Ubiquitin-like modifier-activating (enzyme)
UBC/UBE	Ubiquitin conjugating enzyme
UBD	Ubiquitin D
UBL	Ubiquitin-like protein
UCHL3	Ubiquitin C-terminal hydrolase L3
UFC1	UFM1 conjugating enzyme 1
UFL1	UFM1 specific ligase 1
UFM1	Ubiquitin-fold modifier 1
UFSP	UFM1 specific peptidase
URM1	Ubiquitin-related modifier 1
USE1	UBA6-specific E2 conjugating enzyme 1
USP18	Ubiquitin specific protease 18
VHL	Von-Hippel Lindau
